# What influences communication about retention in randomised trials: a multi-trial, theory-based analysis exploring trial staff perspectives

**DOI:** 10.1186/s12874-022-01708-4

**Published:** 2022-08-25

**Authors:** Taylor Coffey, Eilidh Duncan, Heather Morgan, Katie Gillies

**Affiliations:** 1 Health Services Research Unit, Health Sciences Building, University of Aberdeen, Foresterhill, Aberdeen, AB25 2ZD UK; 2 Institute of Applied Health Sciences, University of Aberdeen, Aberdeen, UK

**Keywords:** Clinical trials, Retention, Theoretical domains framework, Behavioural science, Qualitative interviews, Trial staff

## Abstract

**Background:**

Retention (participants completing a trial) is a persistent, and often under-studied, challenge within clinical trials. Research on retention has focussed on understanding the actions of participants who decide to remain or withdraw from trial participation and developing interventions to target improvements. To better understand how trial staff may influence participants to remain or withdraw from trials, it is important to explore the experiences of staff that recruit and retain said participants and how the process of recruitment impacts retention.

**Methods:**

Two qualitative interview studies informed by the Theoretical Domains Framework (TDF) were conducted with staff involved in various stages of clinical trials. The first set of interviews were focussed on staff perceptions about why participants failed to be retained and what helped to keep others engaged in trials, but also explored more generally what strategies or factors contributed to retention in trials. The second set of interviews were focussed on staff perceptions specifically about the recruitment and informed consent process and how that may influence trial retention. All interviews were analysed using the TDF and assigned to relevant behavioural domains according to perceived barriers/facilitators of the target behaviour. Belief statements were generated, summarising the narrative content of related responses within these behavioural domains. These belief statements were further analysed for themes that captured higher order relationships between separate beliefs within and between behavioural domains.

**Results:**

Twenty-five participants (9 retention staff and 16 recruitment staff) were interviewed. Themes describing the barriers/facilitators to retention broadly, and to communication of retention information at consent, were generated. Four themes on retention broadly and six themes on communication of retention information at consent were identified. Overall, beliefs within all fourteen TDF domains populated these themes.

**Conclusions:**

This study explored staff perspectives on retention and how they interpret their behaviour as contributing to retention success. Perspectives varied considerably but several key themes regarding communication were seen consistently. Specific barriers and facilitators within these findings will serve to guide the design of a behavioural intervention aimed at addressing issues within retention. Findings contribute to a notable gap in the literature on staff behaviour in trials and on retention generally.

**Supplementary Information:**

The online version contains supplementary material available at 10.1186/s12874-022-01708-4.

## Background

Randomized clinical trials (herein referred to as trials) are often considered the foundation of evidence-based medicine [1]. The conduct of trials requires the careful coordination of complex healthcare networks and research teams. However, even the most well-coordinated trials face logistical issues that affect trial outcomes. These can include coordinating teams across multiple sites, differences in site-specific training of staff, and the competing demands of other trials, to name a few. Two of the most persistent challenges when conducting trials are recruiting and retaining participants (i.e., identifying potential participants, enrolling them, and then keeping those enrolled on study until data collection is complete). Methodological research into trial recruitment and retention have been identified as top priorities for the UK clinical trials community [2, 3]. Recruitment has often been the focus of trials methods research, which leaves research into improving retention in need of investment [4–6].

Failures in trial retention can be defined as ‘instances where participants are prematurely “off-study” (i.e., consent is withdrawn or participants are lost to follow-up) and thus outcome data cannot be obtained from them’ [7]. It is estimated that around 50% of trials experience a loss to follow up of at least 11% with some as high as 20% [8]. In fact, some pose that any trial with loss to follow-up over 20% would fail to withstand scrutiny on the strength of their results [9, 10]. The generalizability and internal validity of a trial is at risk from differential loss to follow-up as it introduces bias that can skew effects towards one group or another [5, 10, 11]. Even non-differential loss to follow-up can result in a loss of statistical power and the associated confidence in conclusions drawn from these data [8–12]. Beyond the practical and financial costs associated with replacing those lost to follow-up, ethical concerns need to be considered. If a trial is forced to extend recruitment and follow-up to achieve adequate power, the number of participants that may be exposed to unnecessary risks increases. Those trials unable to reach such power will also be less likely to confidently determine a treatment’s effectiveness, undermining the investment of participants.

Approaches to address consequences of poor of retention have typically involved statistical methods to deal with missing data [9]. Strategies to improve trial retention prospectively have largely focussed on trial participants, with many interventions developed and evaluated but little conclusive evidence on what works, and even less on strategies targeting trial staff [5]. In addition, whether these approaches address perceptions of participants who do not complete a trial is also in question. A qualitative evidence synthesis exploring participant reasons for drop out identified several themes that predominantly have to do with participants’ understanding and/or beliefs about their role in a trial and their “fit” in continuing [13]. It was suggested that not completing a trial may be influenced by inadequate consent processes that fail to set expectations with these participants [13].

For many trials, trial staff are expected to deliver information to potential trial participants during an initial recruitment consultation. This consultation should include sharing of information relevant to retention, such as the ability to withdraw voluntarily, the participant's responsibilities in the study, and the expected duration of their participation in the trial. However, how much of this information is communicated, and whether recruiters prioritise delivering that information, has been put into question [14]. In an analysis by Kearney et al. of patient information leaflets (PILs), only eight trials (16%) made any reference to the importance of patient retention or data collection and no PILs explicitly discussed the problems caused by incomplete data collection [15]. Only 17 trials (34%) described options for partial data collection to help retain patients and this information was not communicated consistently across corresponding trial documents [15]. An investigation into how retention information is communicated verbally during recruitment discussions has found a similar dearth of mentions [14]. Of the recruitment discussions analysed, 79% of them did not include any mention of retention and, among those that did, the conversation regarding retention only occupied 3% of the time in consultation [14]. There is then a need to further assess how recruiters are approaching discussions leading to informed consent that should, ideally, contain information relevant to participant retention.

Retention as a whole is comprised of many separate but interrelated behaviours (i.e., actions of individuals). For participants, behaviours can include returning questionnaires or attending clinic visits. For trial staff, that may include communicating with participants or entering outcome data. Methods from behavioural science can be used to understand what drives behaviour by defining key influences that contribute to that behaviour and how they interact. Those influences can include factors internal to the individual (e.g., motivations, attitudes, or beliefs) and external influences (e.g., environment, resources, or the behaviours of others) [16–19]. The Theoretical Domains Framework (TDF) has been utilised in the context of clinical trials to understand both participant and staff behaviours. The TDF is an amalgamation of 128 explanatory constructs drawn from 33 psychological theories that were deemed relevant to understanding and changing the behaviour of healthcare professionals [20, 21]. It was designed to simplify psychological theories relevant to behaviour change and to make it accessible to those looking to design or evaluate interventions [20, 21]. Newlands et al. used the TDF through qualitative interviews to identify barriers and enablers to trial participants returning questionnaires and/or attending follow-up at clinics [22]. Ellis et al. and Guillot et al. both used TDF-based interviews to assess barriers and enablers amongst clinicians to refer/enrol patients to clinical trials [23, 24]. This current study aimed to use the TDF and explore the behavioural influences on trial staff’s behaviour with regard to retention in trials. Our objectives were to identify which barriers and enablers to retention were relevant to staff within the context of their roles in trials. In particular, we sought to identify those barriers and enablers to retention that exist during the recruitment process and their perceived influence on trial follow-up success.

## Methods

### Specification of the target behaviours

Sufficient specification of the behaviour under investigation is paramount to a successful application of behavioural theory [25, 26]. One of the frameworks that has been used to specify trial specific behaviours is the Action, Actor, Context, Target, Time (AACTT) framework [26].The AACTT framework was used to specify the target behaviours of this study and are presented in Table 1.

**Table 1:** AACTT specification of target behaviour of each interview set

Interview set	Action	Actor	Context	Target	Time
Retention staff	Actions/non-actions that influenced non retention	Trial study staff or trial site staff or clinical staff (e.g., Research Nurses, Trial Managers, Data Coordinators)	Various (e.g., trial office (on the phone, by email, web-based, etc.), clinic (i.e., face-to-face)	All trial participants	Dependent of trial follow-up time points
Recruitment staff	Verbal communication about retention to trial (i.e., attendance at clinic, return of questionnaires, if applicable, ability to stop treatment but maintain follow up)	Trial recruiters	Informed consent discussions	Potential trial participants	Before seeking consent and randomisation

### Participant identification

Two cohorts of trial staff were interviewed in this study. The first cohort, hereafter referred to as “retention staff”, were trial staff members primarily involved in the process of retention (i.e., issuing questionnaires, contacting participants for follow up data, oversight of trial retention activity). These retention staff were interviewed on their perspectives on retention more broadly. The second cohort, hereafter referred to as “recruitment staff”, were trial staff members who were primarily involved in having conversations about trial participation with potential participants. These recruitment staff members were interviewed on their perspectives specifically on discussions of retention information during informed consent. Each cohort was sampled from a different set of host trials, resulting in a high diversity of staff and trial experiences.

#### Retention staff

Ongoing trials with ‘poor’ retention (defined as those with more than 15% missing primary outcome data) were selected from the clinical trial portfolios of project contacts and also through adverts on social media. Trials were either actively in follow-up or had recently completed follow-up procedures. Five trials were selected purposively, all of which were phase III pragmatic effectiveness trials with adults consenting for themselves in non-emergency settings. Further details on trial selection and recruitment are published elsewhere [22, 27].

Trial staff (e.g., research nurses, trial managers, data coordinators) associated with the five host trials (which were set in within urology, frailty and aging, dentistry, and gastroenterology) were invited for interview. One-to-one semi-structured telephone interviews were conducted informed by the TDF topic guide. Verbal informed consent was sought from each participant before interviews commenced. All interviews were audio-recorded and transcribed verbatim by an external company.

#### Recruitment staff

Active trials were identified through known professional networks and social media. Trials could be from any speciality or design but needed to be a clinical trial in which adults consented under their own capacity. Clinical trials were defined as “any research study that prospectively assigns human participants or groups of humans to one or more health-related interventions to evaluate the effects on health outcomes” [28]. Trials needed to be either actively recruiting or have finished recruiting no longer than 12 months from the start of data collection. Trials included those in which recruiters were also tasked with follow-up procedures or in which they were primarily responsible for recruitment/enrolment of participants, but then handed off study follow-up to dedicated trial staff (e.g., a central trial office).

Participants were recruited from both eligible host trials and separately through known professional networks and social media. Those recruited through host trials were invited through an invitation sent via the trial’s central email account by supervising trial staff. Details of this study, along with a participant information leaflet and contact information for the study team, were included in the email. It was stressed to recipients that participation in this study was voluntary but that it was approved by the trial’s steering committee and would not adversely impact their work if they chose to participate or not. Participants recruited through other means (i.e., those not employed through a host trial) contacted the study authors to indicate their interest in being interviewed. A participant information leaflet was forwarded via email and any questions were answered either via email or immediately preceding the interview. Verbal informed consent was taken before the interviews commenced. Interviews were conducted remotely via video call (i.e., Microsoft Teams). Interviews were audio recorded and then transcribed verbatim by an external company. These transcripts were checked for quality and de-identified.

### Data collection

#### Retention staff interviews

These interviews were conducted between 21/02/2019 and 02/04/2019 as part of a project titled “Systematic Techniques to Enhance rEtention in Randomised controlled trials” or STEER, the protocol for which is published [27]. An interview topic guide was developed using guidance on TDF-based qualitative techniques, as well as experience from the study authors in designing similar interviews [13, 14, 22, 27, 29, 30]. The topic guide was refined through group discussion, following AACTT specification of the study’s target behaviour. The topic guide was then piloted through mock interviews, after which it was further refined to optimise wording and flow of questions. The final version of the topic guide is available in Additional file 1. Interviews were conducted by a research fellow (RN) and the initial three interviews were assessed for quality by KG. Ultimately, the purpose of these interviews was to explore staff’s perspectives on why trial participants fail to remain on trial, as well as strategies or factors that promote retention. These interviews were not included in the analysis of the STEER study as their target for intervention development was the behaviour of trial participants, as opposed to staff. Accordingly, the content of these staff interviews presented an opportunity to be used within this current project to maximise its output and efficiency.

#### Recruitment staff interviews

Semi-structured qualitative interviews, informed by the TDF, were conducted for this group between the 24th of May and 19th of August 2021. An interview topic guide was developed using guidance on TDF-based qualitative techniques, as well as experience from the study authors in designing similar interviews [13, 14, 22, 27, 29, 30]. The topic guide was refined through group discussion, following AACTT specification of the study’s target behaviour. The topic guide was then piloted through mock interviews, after which it was further refined to optimise wording and flow of questions. One further iteration of the topic guide was completed after the initial three interviews following feedback from the study team. The final version of the topic guide is available in Additional file 1. Interviews were conducted by the study’s first author (TC) and the initial three interviews were assessed for quality by KG. The interviewer (TC) adapted the order of questions within the topic guide to facilitate the natural flow of conversation.

### Data analysis

Interview transcripts were imported into NVivo qualitative analysis software (Version 12 [31]). Coding guides for each target behaviour were developed using the domains and constructs of the TDF and refined through group discussion. Coding was completed independently by one author (TC) and two transcripts from each interview set were double-coded by another (ED) to assess fidelity of the coding guide and quality of coding. These double-coded transcripts were reviewed during a group meeting to discuss any discrepancies and reach consensus between the coders. Verbatim data were coded into appropriate behavioural domains using the coding guide.

Once data were coded into domains, excerpts within these domains were reviewed to identify emergent beliefs across interviews. These beliefs were summarised into belief statements that captured the core narrative content of these utterances, with the associated domain (e.g., knowledge, skills, etc.) framing the structure of these statements. Belief statements were then analysed for emergent themes that captured similarities between these statements. These themes would provide higher level summaries of barriers and facilitators to the target behaviour that appear relevant across separate, but related, beliefs, and across different TDF domains. Both the belief statements and the resultant themes identified were reached through team consensus, with particular attention being placed on frequency, presence of conflicting beliefs, and the strength of beliefs [30].

For the purposes of a comprehensive analysis, a broad perspective on the target behaviour of retention to trials was adopted, as participants often spoke on the behaviour of others (e.g., other trial staff or participants) than exclusively about their own behaviours. The belief statements that will be presented were thus produced to differentiate the proposed actor(s) involved (e.g., I am confident [self], My colleagues don’t [other staff]).

## Results

The results presented here are reported per the Consolidated criteria for reporting qualitative research (COREQ) checklist. This checklist is available in Additional file 2.

### Participant characteristics

Participant characteristics for both interview sets are presented in Table 2, with further detail provided in the following sections.

**Table 2:** Participant characteristics by interview set; NS = not specified; *Data is number in retention staff; recruitment staff

*Role*	**Retention staff**	**Recruitment staff**	**Self-identified gender**
	*Number (mean time in role, in years)*		
Trial manager	3 (M = 5.7)	N/A	Woman = 2, NS = 1
Trial administrator/data coordinator	3 (M = 5.7)	N/A	Woman = 2, NS = 1
Research nurse/senior research nurse	3 (M = 5.3)	10 (M = 6.6)	Woman = 3;9*, Man = 1
Consultant	N/A	4 (M = 9.1)	Woman = 1, Man = 3
Other research role (e.g., fellow, physiotherapist)	N/A	2 (M = 0.3)	Woman = 2

#### Retention staff interviews

For those interviews involving retention staff, participants represented four host trials. The trials were broadly within urology, frailty and aging, dentistry, and gastroenterology. The host trials included a range of interventions: two host trials were evaluating surgical interventions, one trial a pharmaceutical intervention, and the fourth trial was evaluating alternative monitoring schedules. All four trials used the same method of outcome data collection by requiring participants to return questionnaires through post, with one also requiring the attendance at a clinic visit. Follow-up timepoints ranged from singular follow-up at six months, to multiple timepoints from three to 24 months after participant randomisation. Information on how staff responsibilities were delegated (as reported below for recruitment staff) was not collected for these trials. A total of nine trial staff members were recruited and interviewed, with four members of staff from one trial, two members of staff each for two trials, and one member from the remaining trial. Their roles were: trial manager (*n* = 3), trial administrator/data coordinator (*n* = 3), and research nurse (*n* = 3). Time in their roles ranged from three weeks to 10.5 years (mean 5.4 years). Those that self-reported their gender (*n* = 7) all identified as women.

#### Recruitment staff interviews

Sixteen participants were interviewed, fourteen from across five host trials and two not associated with a host trial, instead identified through social media. These trials were broadly in 1) orthopaedic surgery, 2) urology, 3) sleep medicine, 4) dermatology, and 5) gastroenterology. As mentioned previously, all trials consisted of adults consenting for themselves and the trial outcomes were typically patient-reported but also included safety and economic outcomes, as relevant to their design (additional details on these host trials is available in Additional file 3). Trials varied in the breakdown of responsibilities across local and central sites. Trials 1, 2, 3, and 5 allocated a central study team to facilitate the collection of patient-reported outcomes that required participant input (i.e., questionnaires sent to participants). Recruiters in these four trials were still expected to monitor participants, typically through entering data from medical records into case report forms. Trial 4 tasked staff recruiting to also complete all follow-up (i.e., schedule and conduct follow-up visits and collect outcome data from participants). Participants predominantly identified as women (*n* = 12, 75%) and were employed as a research nurse at varying seniorities (*n* = 10, 62.5%). Other roles included research physiotherapist (*n* = 1), research fellow (*n* = 1), and consultant (*n* = 4). Average length in their roles varied considerably, ranging from six months to 22 years (mean = 6.4 years). Participants were involved in recruiting to a number of trials, with some involved in one to three trials and the highest being 20–25 trials (median = 5).

### Overall findings

In total, 25 participants across the two interview data sets provided their experiences and beliefs about trial retention. Results are presented below within two overarching themes. Within the first overarching theme of “Critical components that comprise retention”, four themes were identified: “Retention is not an equal priority to recruitment”, “Effective relationships are key to retention”, “Communication is the cornerstone to promote retention”, and “A sense of agency informs the belief that what you do matters to retention”. Six themes were identified in the second overarching theme of ‘Verbal communication of retention information at consent’, these were: “Recruiter reflections on their practices and overall trial retention”, “The importance of trying to contribute to retention”, “Being responsive to the individual guides the conversation”, “The practices that guide the conversation”, “Personal experience(s) and its influence on future conversations”, and “Trial-specific and general work-related factors that influence recruiters’ ability to have effective conversations”. The overarching themes and their individual themes are presented in detail below.

### Critical components that comprise retention

This theme recognises that retention is defined not as a distinct action but as a grouping of separate and related actions that contribute to the outcome of retention. In other words, none of these actions by themselves are sufficient to be considered as retention but all can be considered necessary to achieve retention. This overarching theme includes data from across both sets of interviews (N = 25), as both sets of participants offered perspectives that can be included as contributing or restricting efforts to achieve retention. TDF domains are listed in parentheses next to each theme when described below. The belief statements that contribute to these themes are listed in Table 3, along with their associated TDF domain and illustrative quotes for each belief statement.

**Table 3:** Themes describing what constitutes retention and the associated belief statements; RET-00 = retention staff participant ID, REC-00 = recruitment staff participant ID

*Theme*	*Belief statements (and their domains) comprising theme*	*Illustrative quotes for belief statement*
*Retention is not an equal priority to recruitment*	I think that what retention encompasses and why it is important is not emphasized to us (Knowledge)	“That just seems like a huge waste of everybody’s time, you know, we really need to do more to make sure that people understand about retention as much as they do about recruitment. But things don’t seem to be geared that way at the moment.” – RET-02, trial manager
	We do/ do not always know what the alternatives are to full participant withdrawal (Knowledge)	“I did get quite a lot of, especially in the early days, there was quite a bit of grey area for patients withdrawing or simply changing status, or how did we find out, sometimes it was difficult to know if we just found out from a practice that a patient no longer wanted to take part on the trial, what did that mean exactly. And we weren’t sure if we would be able to actually contact the patient to ascertain what no longer taking part to them meant.” RET-03, trial administrator
	I am not sure that how I try to retain participants is effective (Beliefs about consequences)	“R –Do you feel that discussing completing study follow-up with potential participants during recruitment, do you think it makes a difference to retention overall, in your trial? P – I’m not actually sure. No, I’m not sure about that one actually. I don’t know. I’m trying to think of examples where I maybe have in the past, but I don’t know whether that’s been a specific thing that’s give patients on a trial or not.” – REC-06, research nurse
	It feels that some of my colleagues do not consider retention as part of their role (Social professional role and identity)	“I think being upfront with sites because sites are the ones that are recruiting and make them more responsible for retention as well. Or more – not more responsible, making them work with you more because I think there is that cut-off where they recruit and then they think you retain. I think it’s maybe trying to work with sites a little bit more to try – because I definitely think there’s a them and us.” – RET-01, data coordinator
	Training that focusses on retention should be provided to me and/or my colleagues (Behavioural regulation)	“I think it would be useful actually, I think it would be something that would be good for the whole department. I think that perhaps there may be a lack of understanding from some people about exactly what retention is, what it means in terms of the work that we do and just the implication that that can have. I think there could probably be some really good examples given to highlight the difference in what the end result might be from a trial.” – RET-02, trial manager
*Effective relationships are key to retention*	We should be able to establish and maintain effective relationships with participants over time (Skills)	“I guess, as well, part of the retention process is trying to be a sort of a contact point for people, so that hopefully if they have any worries, queries, concerns, throughout their time in the study, they have that central contact point off me as the research nurse. Hopefully, it puts more of a human spin on things for them, and hopefully makes them feel that this is not just them as a number, but that we do see them as an individual.” – REC-14, research nurse
	When we maintain relationships with participants and emphasise the importance of their participation, it contributes to retention (Beliefs about consequences)	“So, there’s all those things going on but there is also that human element of the person who’s giving us their consent. Feeling comfortable with remaining with the trial, feeling that they are valued, feeling that they understand what they are giving us, and what’s happening to them. That’s very important […] that people feel valued and understand why they are important in the context of the trial … I think are important elements of retaining them.” – RET-04, trial manager
	The relationships we establish and work to maintain with participants are crucial to retention (Social influences)	“Well I think probably like a psychological influence if you develop a nice rapport with the patient. A lot of patients I give them… they know my… although I’ve recruited them to the trial, that is my remit finished, that was my remit finished for [HOST TRIAL], everything was done from me collecting data on them rather than me seeing them throughout the trial, but I always make sure that they’ve got my contact number and if they’ve got any issues then they’re free to contact me, but I won’t be contacting them but they can contact me. I think if you develop that rapport with the patient and give the patient that bit of security, that they’ve got your number and they’ve got you as a back up, then I think they’re more likely to complete the trial for you.” – REC-15, research nurse
	I enjoy my relationships with participants and that encourages me to retain (Social influences)	“And then, for me as a person as well, I always think it means… it gives you, as a researcher, the chance to go back to that participant, whether you’re seeing them in person for the follow-up or even it’s just on the phone. But just to know how they’ve got on, how they are, and to maintain that relationship with them. The follow-up allows for that relationship to continue, so that is an additional incentive and that’s a very important incentive for me too.” – REC-07, research fellow
	I am motivated by positive interactions with participants and other staff (Reinforcement)	“Oh, to retain them. I suppose if they’re nice, it helps! It encourages me to retain them because I’m a nurse and I want to move things forward in clinical care. I wouldn’t like to think that we’ve put all this energy into trials and then we didn’t get the answer, that’s why we’re here.” – RET-05, senior research nurse
	Support from my local colleagues and central trial staff improves my ability to retain (Social influences)	“I think I’ve gained… I genuinely feel that I’ve gained a better understanding of that through being faced with the muscular skeletal research unit here. I think because it’s an academic unit and you are working with so many different professionals who are all involved in a research programme, I think it gives you a bigger picture, as opposed to being a recruiter within… my other role, I guess, is more we are delivering studies but a lot of it is focused on recruitment and delivering, I don’t know, the day to day study processes.” – REC-14, research nurse
*Communication is the cornerstone to promote retention*	We should be able to communicate what participation involves early and explicitly (Skills)	“No, I think it’s part of the interview and the consent that they understand there will be follow-ups and how many follow-ups and how long will they take, so when they consent it’s what I call a valid consent, they understand not just the study, but they understand what we want from them for the whole of the study.” – REC-10, research nurse
	When we set realistic expectations with participants about what trial participation involves, it leads to improved retention (Beliefs about consequences)	“But I think it is managing expectations, really. If you’re going to tell people it’s a really quick questionnaire and then they’re going to get 30 pages plus, you know, it does affect their trust in you and their likelihood to want to carry on because they think, well, she said this. But now I’m having this. What’s it going to be like next time? So, yeah, I think it is managing expectations probably be the absolute main thing, really that you need to do.” – REC-03, research nurse
*A sense of agency informs the belief that what you do matters to retention*	We try to accommodate our participants in order to improve retention (Goals)	“And then other things like you know, for me I tried my best to accommodate patients if they weren’t able to attend an assessment, we, and as a team, we tried our best to go back to a practice, especially if the first set of assess… final assessments you know, there hadn’t been a good turnout we tried our best, and level best, to get as many people as we could to return for those assessments.” – RET-03, trial administrator
	I think flexible follow-up options help with retention (Environmental context and resources)	“I think it’s pretty well set up in terms of… from recruitment it’s really quite flexible from being able to recruit patients virtually or face-to-face. In terms of retention, as well, everything’s done via the clinical pathways, so there’s nothing extra that they’re having to come in for, that we’re having to see them for, so because it’s done, we’ll see them at a visit that they’re into see either the surgeon or a nurse specialist. You know, we can catch up with them at that point: we’re not having to invite them in on top of that. And there’s things that we can do virtually as well: we can go over questionnaires on the phone with them, we can post things out, and they can post things back, so I think the design of the trial is quite good and it allows flexibility for patients to be able to sign up and not have that massive commitment where they have to attend hospital several times in the trial.” – REC-06, research nurse
	I think there are changes to study documents that could better emphasise retention to both us as staff and participants (Behavioural regulation)	“I don’t consciously think, “I don’t need to worry about this bit, this is less important,” but I think the fact that it’s further down the consent form probably reinforces… these are the bits you really, really have to make sure go in first, these are the bits that you don’t need to worry about so much. I don’t mean you don’t need to worry about them, I just mean it feels like if you present me a consent form in this order, this is the order of priority as determined by the study designer. One thing that I do think about follow-up is that of all of the statements on the consent form, the follow-up is often the most convoluted.” – REC-13, research nurse
	I think questionnaires are confusing/cumbersome for participants (Environmental context and resources)	“I mean, comparing [HOST TRIAL] to some other studies I’ve had which have been 30 pages of questionnaire on one side injury and then 30 pages on the same limb but on a different side. And they would get a lot of reminders to fill out these questionnaires. There would be a lot of questionnaires and I think you lost patients that way because they didn’t want to fill out the questionnaires it was boring. They were doing, you know, where there’s [HOST TRIAL] it’s not having that – fewer questions is definitely helpful.” – REC-03, research nurse
	I think there are ways for us to deliver questionnaires to participants that would make it easier for them to complete and return them (Behavioural regulation)	“Yeah, but with [HOST TRIAL] all they had to do was fill in maybe ten pages of questions, tick box, the envelope was sent to them and they just posted it, they didn’t need to pay any postage, they got a voucher if they completed the questionnaires. So [HOST TRIAL] really you know, yeah, was a good study to recruit to and a good study for retention.” – REC-15, research nurse
	I think participants’ competing priorities from ‘real life’ context can be a barrier to retention (Environmental context and resources)	“Plus, I think all of our trials are trauma studies so they’re not really the best situation to be giving somebody a lot of information. They’ve just had an unexpected injury, they’re probably in quite a lot of pain, probably also had some painkillers which might mean that they’re not thinking as straight, so I think there’s a lot of times where, actually, we’re giving these people all this information. I do think that often, the follow-up information can kind of get lost between everything else, because they’re more thinking about, ‘What treatment am I going to have right now?’ rather than, ‘Oh, you’re going to ask me some questions about it in a year’s time.’” – REC-02, research physiotherapist
	It feels like our participants’ motivation to be in our trial, not necessarily how motivated we are, predicts whether we can retain (Intentions)	“Often, I’ve found participants are keen on the intervention, but they’ve been put off by the intensity of the work… of the burden of the follow-up – the questionnaires, interviews. Not so much the visits or the appointments, but when they’ve got to do things, like complete things, and send them back to you, or sit through an interview with you and things, and that can… I think if someone’s going to take part in, or… sorry, I think if someone’s going to decide to leave, they’re going to leave that process anyway irrespective of the researcher.” – REC-07, research fellow
	Retention can be stressful/frustrating for me (Emotion)	“I feel happy if I can retain participants in trials. If we go to the effort of keeping patients in trials and they don’t turn up for visits, that makes me quite angry. It has a knock-on effect with every other trial that I do. I feel it should be reiterated to patients that it’s a hospital and they can’t just not turn up for appointments.“ – RET-05, senior research nurse

#### Retention is not an equal priority to recruitment (Knowledge, beliefs about consequences, social professional role and identity, and behavioural regulation)

What retention encompasses, and what leads to “good” or “bad” retention, seems to be less well understood compared to analogous processes within trial recruitment. Staff demonstrated an awareness that recruitment is often given a higher priority to retention in the way it is operationalised and incorporated into the specific roles within a trial and the research culture present at an institution. On a practical level, this can mean that staff are not sufficiently informed on the strategies that may be available to them to promote retention. For example, staff indicated that full participant withdrawal is often defaulted to, even where alternative means of participation are available that would allow outcome data to be collected in some form. Staff were also unsure of how effective existing strategies of retaining participants were.

Staff also indicated that there were tensions among research teams regarding who actually carried responsibility for retention. Roles may be more strictly demarcated in some trials or institutions, leading to a less holistic view of one’s contribution towards a trial’s success. When considering possible ways to ameliorate both these practical and cultural issues within trials, training that focused on retention was suggested. This included raising a general awareness about the importance of retention and the implications of poor retention, redefining roles within the trial to encourage a synergistic view between separate teams, and instruction on retention strategies available to the team and the evidence supporting those strategies.

#### Effective relationships are key to retention (Skills, beliefs about consequences, social influences, and reinforcement)

Trials were acknowledged as an inherently interpersonal endeavour and the need to nurture such relationships was viewed as fundamental to their success. A key skill that was emphasised by staff was the ability to form and maintain effective relationships with participants. These relationships were believed to precipitate a shared sense that participants are partners within a trial and participant contributions should be acknowledged. Staff often pointed to these interpersonal elements as potent incentives that motivated them as trial staff to retain. They emphasised that being able to have such relationships was an important aspect of what they enjoyed about their role in trials. Positive relationships with other staff members were also seen as contributing to retention success. Open and collaborative relationships between members of local teams and trial colleagues in other centres fostered beliefs in staff’s ability to engage effectively within their own roles and respond to challenges.

#### Communication is the cornerstone to promote retention (Skills, beliefs about consequences)

Staff presented strong beliefs in the efficacy of communication in their roles and its influence on retention. Staff who had a more active role in retention advocated for the importance of participants receiving adequate information about follow-up during informed consent. There was a belief present amongst these staff members that those recruiting may not be doing so and that those tasked with retention were facing the repercussions of this. The recruiters interviewed (who were from separate host trials from the staff mentioned above) echoed the importance of communicating follow-up information in their consent discussions. Many emphasised that it is essential that they do so, but some did admit they may not do so to a degree that is effective.

Expectation setting was one of the main goals put forward by staff as to why communication of follow-up information with participants about what they will be doing as part of the trial is essential. At consent, and throughout the study, it was advocated that staff help participants to understand what is expected of them, how the trial differs from usual care, what the trial procedures entail, and how often they occur and how long they take to complete. It was believed that, by not doing so, participants would be unprepared or otherwise dissuaded from continuing their participation.

#### A sense of agency informs the belief that what you do matters to retention (Goals, environmental context and resources, behavioural regulation, intentions, and emotions)

Perhaps one of the most diverse themes, in terms of the breadth of opinion, was whether staff believed they had any substantial impact on retention. Predictably, staff who had less of a direct role in follow-up (i.e., were recruiters) presented with less confidence that they could influence retention. However, even amongst those staff tasked with follow-up, one’s ability to influence retention seemed to depend on a perceived sense of control over those outcomes. Staff often discussed the importance of trying to accommodate participants to improve retention. This included adapting ways of working, finding suitable alternatives to collect data, and generally to remain flexible. This staff flexibility was facilitated by the flexible follow-up options that were allowed within a trial. The ability to make necessary changes to the follow-up schedule, location, or procedures in order to accommodate the participant was noted as particularly effective in promoting retention.

Certain aspects of trial design appeared detrimental to staff’s confidence in their ability to retain. It was often mentioned that the design of particular documents could be revisited to better promote retention. This could be aspects of the consent form to emphasise follow-up, to both staff and potential participants. The length or complexity of questionnaires was also described as a barrier. Staff believed that participants were lost when questionnaires were unnecessarily long or contained questions that were not relevant to them or their allocated treatment. Questionnaires that were only as long as needed, formatted to allow simple answers, and easy to return (either through electronic means or pre-paid postage and envelope provided), were advocated to improve retention. Potential changes to how questionnaire data are collected were also suggested. This included translated versions in areas of non-native English speakers or the option to complete questions over the phone with staff.

Where staff appeared most unclear on their relative influence often had to do with factors intrinsic to their participants. Participants’ competing “real life” priorities were often cited as detrimental to retention. It was evident that these competing priorities were not always offered by participants as reasons for dropout and that could lead to frustration for staff who cannot link their efforts to retention. Similarly, it appeared to frustrate staff when their motivation to keep someone engaged in a trial was not met with similar motivation from the participant. The motivation of the participant was described as one major factor in retention that was relatively outside the ability of staff to influence.

### Verbal communication of retention information at consent

The results presented below represent the perceived barriers and facilitators to communicating follow-up information to potential participants at the time of consent and is thus restricted to the second set of interview participants involved in recruiting (*n* = 16). These interview participants were primarily tasked with recruiting to trials, but some were also involved in collecting follow-up data, to varying degrees. Themes and their associated belief statements, along with illustrative quotes for each belief statement, are provided below in Table 4.

**Table 4:** Themes describing the overarching theme of communication of retention information at consent and associated belief statements; REC-00 = recruitment staff participant ID

Theme	Belief statements (and their domains) comprising theme	Illustrative quotes for belief statement
Recruiter reflections on their practices and overall trial retention	I consider retention to be… (Knowledge)	“So, retention on to a study would be to achieve all of the follow-up that was included as part of the study, and the reason for that is because of the outcomes and answering the question. Yeah, follow-up, so retention is completing the follow-up and completing the study.” – REC-04, senior research nurse
	I know what follow-up involves in my study and how I talk about it with participants (Knowledge)	“It’s really just ensuring that the study that you’re recruiting them to, that you’ve got a good understanding of it, so that you can explain to them… you know, “This is your level of involvement that we need” or, “There’s not that much involvement. We do this at the screening visit, and we collect your bloods, and we collect some data, and that’s it. We don’t need anything else from you.” There might be some follow-up questionnaires maybe from the study centre, but in terms of us, there’s not really much else involvement. Again, it always just comes back to the same point, I think really, just about being informed.” – REC-06, research nurse
	I am aware of retention in my study and what we do to ameliorate issues (Knowledge)	It [dropouts] happens, in honesty I’ve not picked this up as a problem, and in a way the main counselling I do with them is at the time of consent really. After that, because they get sent questionnaires directly by the trial office, and I suppose the trial office will let us know if they are struggling to get a hold of a patient, but we’ve not had that experience to be honest, either because the trial office has not told me, or because they have been successful in chasing up my patients. I mean I see the patients and I chat to them, and I say, ‘have they sent you anything’ and they say ‘yes, they sent me a questionnaire and I filled it in’, but we have not had a problem that way as far as I know.” – REC-12, consultant
	I am aware of research about retention (Knowledge)	“I guess we’ve got some maybe some slightly innovative ways that have been developed through this unit to try and aid retention. We do send things out like a reminder card prior to a questionnaire, so that patients are aware that that’s coming out soon. And I’m sure this us all evidence-based to ensure retention.” – REC-14, research nurse
	The training I received did not typically cover retention (Skills)	“I’d say most of the training that I’ve had has been quite practical, so I’ve done an online course of approaching patients which is called Granule, but that’s more about the randomisation, explanation about how you kind of approach… keeping equipoise within both of the treatments. It didn’t really go much into follow-ups.” – REC-02, research physiotherapist
	I am confident in my ability to discuss follow-up during my recruitment discussions (Beliefs about capabilities)	“Yes, I wouldn’t sign up to the trial if I wasn’t comfortable discussing it [follow-up].” – REC-12, consultant
The importance of trying to contribute to retention	It is necessary that I discuss follow-up (Intentions)	“R—So when you go into those discussions do you always intend to discuss retention with the participants then? P—Yeah, absolutely, yeah R—Does that vary or change over time or is it about the same? P—No, it’s part of the, in my opinion it’s part of the consent process. If you’re consenting a patient on to a research trial then you have to discuss retention.” – REC-15, research nurse
	I think consent is only valid if participants have received an adequate amount of information about follow-up (Beliefs about consequences)	“It’s [talking to potential participants about follow-up] part of valid informed consent, you know? You’re not receiving consent just to say, “Yes, you can do this operation,” or, “Yes, you can give me this medication.” The whole study is what you need to take valid informed consent for, and so ensuring the participant is saying yes to all the parts of the study, it’s as important to have their consent to keep seeing them for a year, as it is for them to have the original intervention.” – REC-13, research nurse
	I would hope that my conversations about follow-up have an effect on retention, but I am not sure they do (Optimism)	“R- Do you feel that discussing completing study follow-up with potential participants during recruitment makes a difference to retention overall? P– I’m not actually sure. No, I’m not sure about that one actually. I don’t know. I’m trying to think of examples where I maybe have in the past, but I don’t know whether that’s been a specific thing that’s give patients on a trial or not.” – REC-06, research nurse
	Knowing that my work has contributed to retention and, ultimately, to a successful trial motivates me (Reinforcement)	“I think I mentioned earlier, it’s always in the back of my mind, and I try to bring it into my approach that we need to… that there is this follow-up and if they agree to take part in a study, we’ll need to see them again at these different time points. If they agree to participate and then if they agree to the follow-ups as well, that’s definitely an incentive because that’s what we wanted, and that just looks really good in the study that we’ve had someone who’s been engaged throughout the process at all the different timepoints.” – REC-07, research fellow
Being responsive to the individual guides the conversation	I think seeing participants as individuals can drive your approach to be tailored to them (Social influences)	“If something works well then carry on using that to get patients recruited and be on the study. Also, the other side, if I feel that I’ve done something that hasn’t worked quite as well that time because the patient didn’t receive it in the way that it was maybe meant or whatever, I think that side of it is all good to learn from. No patient is the same, so each time you go in you might have a way of doing things, and I think doing lots of different studies is good in one way because you’re not just doing one study where you’re saying the same thing all the time, you’re jumping from study to study and seeing different patients for different things. I think it keeps you on your toes a little bit more because you’re not just getting into a way of discussing a study with a patient, you know, it’s not the same thing every time. It’s good to see the patient as an individual and talk through things and let it be quite fluid.” – REC-09, senior research nurse
	My role as a recruiter is to be transparent with participants about what study participation actually involves (Social professional role and identity)	“R—So how does talking to potential participants about follow up and completing the study how does that fit within your role as a recruiter? P – It’s completely in my role as a recruiter because you don’t want to recruit a patient to a trial and then they don’t follow… then they don’t [complete] the follow up. So it’s hoping when you’re recruiting a patient that the follow up and what’s expected of them when you’re recruiting them, the patient needs to know what’s expected of them. So that’s definitely a conversation, retention and follow up.” – REC-15, research nurse
	You need to be able to set and manage expectations about follow-up with participants (Skills)	“In terms of the follow up it’s explained very quickly on the video. But we do tell them that the questionnaire that they’re completing at that time it’s no more arduous – the follow up questionnaires are very similar to the questionnaire they’re dealing with at the baseline. So they have this idea of what’s expected of them because I think managing expectations is really key in this.” – REC-03, research nurse
	I try to highlight to participants what their commitment to the trial actually means for them (Goals)	“That’s important to highlight to participants, to say that this is a long, ongoing trial, but to make it clear what the actual involvement is going to be, so is it just going to be a questionnaire through the post at three, six, twelve, and then twelve-monthly for five years, for example. Are participants going to have to physically attend a hospital or a trial site? How much time is involved in each of these follow-ups, and generally, especially for PROM studies where there are questionnaires being sent out, it’s not a very arduous workload. But, for example, a CTIMP study might involve physically coming into the hospital every six months for five years.” – REC-13, research nurse
	I present enough information about follow-up to inform participants, but not so much that I overburden (Skills)	“No, I guess sometimes if it’s an intense follow-up, I guess that’s perhaps the time that it can perhaps be slightly more challenging in the way that you present it. If there’s a lot of follow-up required from it, the patient may not want to be involved for that very reason but you need to make sure that they’re aware of everything, but equally not scare them off because of the quantity.” – REC-04, senior research nurse
	I think it can be overwhelming to participants if we provide them too much information about follow-up (Beliefs about consequences)	“I think there is a danger of getting swamped down in follow ups. And I think a lot of research and recruitment is how you present it to the patient. And I think there is a drawback in getting swamped down. […] I think you can overwhelm a patient with information. And if you’ve got somebody who’s in pain and who just wants their [CONDITION FIXED] but wants to do the study and you’ve gone through everything. You still need to mention follow ups, but I think it’s more a case of, you can say, by relaying exactly – you could go too much into detail. And I think that can be quite overwhelming. But also, I think it’s important to think that just because I don’t think these follow ups are overwhelming doesn’t mean other people won’t.” – REC-03, research nurse
	You need to be able to assess whether a participant’s taken that follow-up information onboard (Skills)	“I think every approach is different because you’ve got to look at your setting and your environment and whether it’s appropriate for the person that you’re speaking to as well, so I try to gauge their circumstances, whether they’ve got capacity as well to understand what I’m saying, if I’ll need a consultee, or if it’s even appropriate to talk about research now: have they had some bad news; are they unwell, and all those sorts of things.” – REC-07, research fellow
	I am not always confident that I have communicated follow-up effectively as it is difficult to assess a participant’s understanding (Beliefs about capabilities)	“I think there’s some people who really can take that [follow-up discussion] on board and they’re like, “Yeah, no problem, that’s fine,” whereas I think for other people, thinking about that is just too much at that time. Then, obviously, once they’re in the study, then they’ve already kind of signed up for it. Even if you’ve explained it to them and you’ve gone over with it a lot of times, I think it can be quite difficult to know whether they’ve actually really taken that onboard. We spend quite a long time with a lot of these patients, we’ll be with some of these patients for an hour, probably, so I think a lot of people can get almost a bit frustrated with all of the information that you’re trying to give them and they almost kind of get a bit saturated by it.” – REC-02, research physiotherapist
	I try to make participants aware of the support they will have throughout the study and that we value their participation (Goals)	“I always explain to them that, you know, even though those… you know, we only see them every six weeks, that their health and safety is really important and I always encourage them to, you know, even if they think it’s minor, I would rather them contact me and me reassure them than them suffer in silence. So, they’re always given that kind of reassurance that they’ve got one person that they can count on…To approach, and I try and be as open as possible to make them feel comfortable and so that they know, you know, I’m here to work with you and help you, and I also stress that, you know, doing a research trial is complimentary to their routine NHS care, and that usually makes them feel quite good as well because they think oh, okay, it’s complimentary and it’s not going to affect their NHS care, so I do try and give them that kind of reassurance and make them feel like they’ve got, not like a friend, but someone that they can confide in and someone that they can be close to, and you know, the fact that we’re always there for them, it’s not like booking a GP appointment, they really like that because they feel like they’re being looked after, so yes, I tend to do that with them.” – REC-16, research nurse
	I try to emphasise the voluntariness of participation and their right to withdraw (Goals)	“R—is there anything else that you normally present during those discussions, that you feel falls under that definition, that you can think of? P – Not particularly, other than the fact that you would be explaining to them that: “You’ve got a right to withdraw at any time, that you can decide at any point at all to withdraw from the study.” There’s nothing really that I’m probably going to be saying because it’s a bit of a grey area and it can overlap into the… sort of almost influencing someone into staying… coming onto a trial and then staying on a trial. Whereas, I think, it’s important to try to be impartial. It’s difficult. It can be really difficult. And there’s examples… you see examples of clinicians, I suppose maybe almost trying to influence patients, in a way, and it’s maybe not intentional, but I think, for me, I really do try and remain impartial and just really let them know that, “It’s entirely your choice, and then obviously, when you’re on the study, staying on the study, again, it’s entirely your choice. You can withdraw at any time.” – REC-06, research nurse
	I am concerned about participants feeling coerced into a trial and I work to remain impartial in my conversations (Social influences)	“It’s just really important to emphasise to the patient that they’re under no pressure, that a lot of patients you know, they think they have to commit to the trial because the doctor’s asked them. So I just like them to know because if the patient feels under pressure and they don’t really want to do the trial, if you recruit them then they’re never going to complete the questionnaires and be retained in the study.” – REC-15, research nurse
	I typically feel comfortable discussing follow-up, but it can vary (Emotion)	“R—How do you feel when discussing follow-up with potential participants? P – I don’t really have any feeling on it. It’s part of the study so it’s one of those things that we have to do, yeah, it’s just normal to talk to them about it R – Yeah. Ever frustrating or stressful or anything like that? P – No. I think, you know, if the patient is understanding what you’re saying, then all is fine. Yeah, I’ve not had anyone where I’ve actually felt frustrated by talking about the follow-up.” – REC-09, senior research nurse
	A potential participant’s health can impact on what I think is appropriate to talk through with them at consent (Environmental context and resources)	“And with [HOST TRIAL] in this hospital, you kind of get a small window because it’s usually the morning before surgery. They’re hungry, they’ve not eaten. They’re usually going through A&E. They’ve had a lot of information given to them about their [CONDITION], as it is. They’ve got to then adapt to the idea that they may not be [recovered] for six weeks. That they won’t be driving for the foreseeable. It’s a big life changing injury for that point in time. It might not be further on but for this point in time it’s quite life changing. And I think that you’ve just got to – there is ways to present it. And it’s a trial-and-error thing and what one patient wants to hear or needs to hear isn’t the same as another patient. So it kind of like, you’ve got to tailor it to who your audiences is almost.” – REC-03, research nurse
The practices that guide the conversation	I prepare for the discussions, typically by reviewing documents (Behavioural regulation)	“I think normally I give myself a minute just to go and read through everything again and remind myself, and I’ve got all my paperwork. It kind of prompts you and then you’re okay.” – REC-01, research nurse
	I have a mental checklist for what I want to discuss (Behavioural regulation)	“I’ve gone through the conversation in my head, and those are things that I would always mention, or they are the things that I would be asked about, so over the years I’ve kind of drafted a mental script of the things that I would like to mention that I think are important out of the consent process.” – REC-07, research fellow
	I use certain trial-related documents to guide my conversations (Environmental context and resources)	“Usually, there’s part in the information sheet there would be… if it’s going to be a trial that’s going to go on for a lengthy period of time and that’s a lot of patient involvement, there usually would be a table of events normally and an information sheet. You’d be able to go through that with them and ensure that they knew the level of participation that’s required from them.” – REC-06, research nurse
	I use tools like the participant information leaflet to prompt me in discussing certain aspects of follow-up (Memory, attention, and decision processes)	“No, I think that’s part of our normal spiel to patients, to talk about the follow-up. The bit that I find hard is remembering how many weeks or months each one is, but when I go to talk to the patient, I’ll always either check it before or I’ll read it from the patient information sheet when I’m there with them, just to make sure what I’m giving them is accurate information.” – REC-02, research physiotherapist
Personal experience(s) and its influence on future conversations	My confidence in discussing follow-up is affected by how much experience I have (Beliefs about capabilities)	“Yeah, I feel quite confident doing that [discussing follow-up]. Yeah, I do that regularly so I think that comes with the confidence, doesn’t it? The more you do it, the more confident you feel with it, yeah.” – REC-04, senior research nurse
	The methods I use to approach follow-up with participants have developed over time (Skills)	“I think the longer that you’ve worked on a trial, the easier it [discussing follow-up] becomes because you get used to the patients that have already been recruited. Sometimes they might have withdrew and gave some reasons why, so you begin to adapt your language almost, and your script that you go through with patients to ensure that you try to reduce withdrawal rates.” – REC-06, research nurse
	Difficult prior experiences with participants affects how I approach my current discussions (Reinforcement)	“R – So, do you always intend to discuss your intention when you talk to participants P – Always, it’s a lesson I learned very early on in my career in research! R – Okay, and what was that, through some negative experiences or you know… P – No, it was just I remember we had a participant who didn’t really understand that they had to complete all the study procedures which I thought was very odd considering they signed the consent form, and they said, ‘but you said I could withdraw at any time’, and I said ‘yes, but by signing the consent form you did actually commit to the study’…And, yes, so I’ve had some odd ones over the years and just make it a point of, you know, definitely going through that, so it’s just little things that I’ve learned throughout my career.” – REC-16, research nurse
	I reflect on my discussions, either by myself or with the help of colleagues (Behavioural regulation)	“We’ve also tried with a few more of our junior members of staff and getting them to self-reflect on it with somebody else there, so they’ll approach a patient with, say, me watching, and then afterwards we’ll have a discussion and I’ll be asking the person that approached them, “How did you think it went? Anything you thought you could have said better or done better?” etc.” – REC-02, research physiotherapist
	I typically do not receive feedback on how I can have effective conversations (Reinforcement)	“I guess we probably all do it in a slightly different way, but having said that, it might be quite challenging to be able to get that feedback because, often, you are in a clinic room and there isn’t somebody else around to listen.” – REC-04, senior research nurse
	As I have gained experience in trials, I have found it easier to remember what to discuss about retention (Memory, attention, and decision processes)	“R—So is remembering to talk about follow-up, is that something that’s difficult or easy to do, do you think? P – I think it’s easy. I think because we know the studies and, I suppose, because we do the follow-up, it’s very much part of what we would be doing and part of the study for us. So yeah, I think mentioning the follow-up is just part of it. You would normally… in conversation even, “I will next see you on the ward with a questionnaire,” or, “I will be posting this one out to you at this time point,” and explaining a little bit, maybe elaborating. “I’ll be sending a return addressed envelope for you to post it back to me.” Kind of talking a little bit more about it. I guess because we do that follow-up, we have that insight into it so yeah, it’s just part of the journey.” – REC-04, senior research nurse
Trial-specific and general work-related factors that influence recruiter’s ability to have effective conversations	The design of the trial affects how I discuss follow-up (Environmental context and resources)	“A&E studies, depends what we’re looking at but for things like PROMS studies, it makes a lot more sense or it’s easier to incorporate follow-up into the conversation because we’re looking at outcomes. We’re explaining to a participant that, actually, “These are patient-reported outcomes so we need you to report your outcomes,” and that’s logical. There are some studies that are, “Actually, we’ve only got one follow-up time point and that’s us contacting you in a year’s time to see how you’re getting on.” It’s quite easy to incorporate that into a short conversation, so thinking about maybe a study that looks at different ECG machines, doesn’t actually impact on the participant very much.” – REC-13, research nurse
	My confidence in discussing follow-up is affected by how intense/frequent follow-up is (Beliefs about capabilities)	“We talk through the whole of the schedule of the study when we’re giving out the information. I also go through all of the… you know, what we’re asking them to do as part of the study, so along with being randomised to different arms, we talk about the follow-up looks like. I think with [HOST TRIAL] it’s slightly easier in some ways because the follow-up was them coming and having their treatment and me looking up the notes, rather than having to do a lot of stuff back and forth with clinics.” – REC-09, senior research nurse
	The volume and complexity of information I need to cover about a trial can make it difficult to remember everything I might need to discuss (Memory, attention, and decision processes)	“R—Is remembering to talk about follow up and completing the study, is that difficult or easy to do? P – Oh it’s easy R – Yes, so you don’t have any issues kind of remembering what you need to talk through? P – No, never, you know with [HOST TRIAL] it’s quite easy, you know, there isn’t a lot of commitment to the follow up so it’s quite an easy… a fairly easy conversation, I’ve got other studies that are way more involved, and it becomes a bit of an issue, so yes, [HOST TRIAL]’s probably one of the easier ones to have that conversation with.” – REC-16, research nurse
	I have other pressures in my job that compete for my attention and memory (Memory, attention, and decision processes)	“R—Is remembering to talk about follow-up and completing the study, is it difficult or easy to do? Is it hard to remember what you need to through or anything like that? P – It can be, especially if you’re in the middle of thinking about another study and then they maybe let you know that somebody suitable can you either come down and see them or give them a ring, and then you feel like you’re jumping… I know, sometimes I’m like, “Oh, my goodness, which study am I working on just now?” – REC-01, research nurse
	If I am under pressure from other work responsibilities or lack of time, it can impact on my discussions (Environmental context and resources)	“I always mentioned that there'll be follow up protocols, questionnaires to fill but whether to the extent of the details which I go with particular patient may vary depending on the time pressures and whatever, just what day is it. So I think I mentioned treatment with everyone but the extent is different.” – REC-05, consultant
	My role as a recruiter is determined by who makes the initial approach (Social professional role and identity)	“R—So how does talking to potential participants about follow up and completing the study how does that fit within your role as a recruiter? P – It’s completely in my role as a recruiter because you don’t want to recruit a patient to a trial and then they don’t follow… then they don’t [complete] the follow up. So it’s hoping when you’re recruiting a patient that the follow up and what’s expected of them when you’re recruiting them, the patient needs to know what’s expected of them. So that’s definitely a conversation, retention and follow up.” – REC-15, research nurse

#### Recruiter reflections on their practices and overall trial retention (Knowledge, skills, beliefs about capabilities)

The range of definitions of retention volunteered by interview participants was broad and varied in the specific detail given. Some participants, notably the consultants, had concise descriptions that defined retention as a trial participant completing follow-up through the associated primary outcome measure. Others offered comprehensive descriptions that included their own responsibilities, those of the trial participant, and why retention is important for a trial, along with the aforementioned completion of follow-up data.

Interview participants were predictably knowledgeable on the follow-up procedures and schedules of their trials, even when they were not directly tasked with that follow-up. They were able to give examples of how they believed they communicated this knowledge during their consent discussions and were typically confident in their ability to do so effectively. There was also a general sense that recruiters attempted to stay informed on the overall progress of the trial and rates of retention, including what strategies were implemented to ensure success in retention. However, some did admit to an unawareness of any issues in retention. They attributed this to a separation in their role from follow-up and conceded this did not mean such issues were absent but rather that they had not been brought to their attention. When asked about what was known generally to drive retention in trials, some participants referenced research on retention, or lack thereof, whilst others mentioned analogous research on recruitment. This was echoed when discussing the training that they had received in trials. A subset believed the training they received to not have covered anything specific to promoting retention. Alternatively, some answered affirmatively that they had been trained to promote follow-up but either did not offer detail on what that meant (e.g., whether strategies were discussed) or seemed to lack confidence in their answer. Those who did offer detail on their training often cited known courses in good clinical practice (GCP) offered by the National Institute for Health Research (NIHR) or other sponsors.

#### The importance of trying to contribute to retention (Intentions, beliefs about consequences, optimism, reinforcement)

Regardless of their training, recruiters nearly universally believed their role within the trial pathway to be important to retention. Often, they believed discussing follow-up was necessary in their consent discussions and that consent would only be valid if trial participants received an adequate explanation of that follow-up. There did not seem to be a clear consensus amongst recruiters how much of an impact their discussions of follow-up had on retention. Some expressed optimism that it would, whilst others were unsure or felt they had no impact. For those who felt they had some degree of impact on overall retention, there appeared to be a sense of professional pride that motivated them to have these follow-up discussions. They acknowledged the rewarding aspect of feeling as if one has contributed to the success of the trial through their own efforts.

#### Being responsive to the individual guides the conversation (Social influences, social professional role and identity, skills, goals, beliefs about consequences, beliefs about capabilities, emotion, environmental context and resources)

Recruiters are acutely aware of the human aspect of trial recruitment and moderate their discussions with potential participants accordingly. The idea that “no two approaches should be the same” was recurrent throughout the interviews. Recruiters often saw their role was to be transparent with a potential participant. They felt that they needed to be able to set and manage expectations about follow-up with participants from the time of consent. This was done by trying to highlight to participants what their commitment to the trial means for them as an individual and how that contributes to the larger success at the site and the trial as a whole. That included being pragmatic in discussing the follow-up appointments and procedures, so participants are fully informed and prepared for a certain level of involvement on their part. However, this information needs to be delivered with respect to the idea that these consent conversations can involve the dissemination of large volumes of information. Recruiters often felt the need to balance their conversations so that this level of information did not become burdensome to participants, especially at the cost of dissuading them from considering participation.

In order to have this balance, it was emphasised that recruiters should be able to assess a participant’s level of comprehension. Recruiters often said that they had to incorporate real-time assessment of a participant’s understanding of follow-up, along with the other necessary aspects of trial participation. This skill was admitted to be challenging to develop and those who were unsure of their ability to make these assessments were less confident in the effectiveness of their consent discussions. These consent discussions were said to be challenging at times, particularly if one was approaching a potential participant when they were unwell. The limitations of an individual’s attention and memory when unwell were cited as reasons recruiters may feel it inappropriate to cover all aspects of follow-up and instead prioritise what is necessary and deemed relevant to the potential participant.

Recruiters were also concerned about the implicit pressures that some individuals may feel to participate in a trial. In difficult contexts, like life-changing injury or chronic illness, recruiters described the need to be further cognisant of their potential influence and present trials impartially. Indeed, consenting under such potentially coercive circumstances was believed to be not just unethical but also lead to poor retention. In order to ameliorate potential pressures, and as an essential point to convey regardless, recruiters sought to emphasise that the trial was voluntary and that they were free to withdraw at any time. Additionally, they often advocated the support available to participants of a trial. The care received in a trial was promoted as being complementary to their typical care, with value added in the research team’s attentiveness and appreciation for the participant’s contributions to the trial.

#### The practices that guide the conversation (Behavioural regulation, environmental context and resources, memory, attention, and decision processes)

Recruiters reported a range of ways to prepare for their consent conversations. They reported reviewing trial documents, like the protocol or participant information leaflet. These same documents were often used during the conversation itself to guide the flow of discussion. Recruiters also mentioned having “mental checklists” for the information they want to discuss. More often, these mental checklists appeared to be a product of experience, potentially freeing up attention resources to be re-directed at responsivity towards the participant.

#### Personal experience(s) and its influence on future conversations (Beliefs about capabilities, skills, reinforcement, behavioural regulation, memory, attention, and decision processes)

As alluded to above, experience seemed to be linked to an ability to remember more easily what to discuss about follow-up during consent. Recruiters often mentioned that experience in their role serves to define their confidence in discussing follow-up and their preferred methods to approach those discussions. These methods appear to be trialled and refined through self-reflection on their behaviour and its outcome. Reflection with colleagues was mentioned less frequently, as the solitary practice of recruitment does not afford such opportunities. This reflection could often be precipitated by difficult or negative prior experiences with recruitment conversations. In some cases, recruiters were able to identify issues within the consent conversation immediately after and integrate these reflections. In other cases, issues did not become apparent until later in follow-up when staff and participant conceptions of trial commitments conflicted.

#### Trial-specific and general work-related factors that influence recruiter’s ability to have effective conversations (Environmental context and resources, beliefs about capabilities, memory, attention, and decision processes, social professional role and identity)

The interviews identified several factors outside the control of the recruiter that have a notable effect on their perceived ability to carry out recruitment conversations that include discussions of retention. The design of the trial was mentioned often as having considerable influence on how these conversations are structured. Those that are focused on patient-reported outcome measures, as opposed to safety/efficacy etc., align the scientific priorities of the trial with the expectations required from participants. This seemed to facilitate retention discussions as recruiters feel they do not have to balance the abstract goals of trial outcomes with the treatment priorities of the participant. The relative complexity of follow-up also, understandably, impacts on recruiter’s confidence. A trial that presents frequent and/or invasive outcome measures are considered more difficult to “sell” to a participant. Those trials with more involved follow-up place strain on the memory/attention capacity of recruiters (and participants), along with straining the time limits of the consultation. These time limitations are further complicated by the competing work pressures faced by recruiters. And, finally, the extent of recruitment conversations is subject to who in a research team makes first contact with a potential participant. Some recruiters start their recruitment process with a participant who has first met with a consultant. These consultant conversations vary in their content, but typically focus on the treatment pathway, with less attention paid to the trial pathway. Recruiters then pick up this aspect of the conversation. In contrast, other trials are designed in such a way that recruiters have initial contact with a potential participant and thus a higher degree of control on the extent of trial-relevant communication.

## Discussion

This study has identified key perspectives from trial staff on the behavioural influences to trial retention at the point of initial recruitment discussions of informed consent and more broadly. These perspectives come from both staff involved in retention directly and those more involved with recruitment. By drawing on the experiences of a wide range of trial staff, in role and in tenure, we have expounded on the complex interplay of behaviours important for recruiters, their colleagues, and trial participants.

The themes generated fall broadly into two overarching themes, those relevant to the full range of staff roles interviewed and those specific to the recruiters interviewed. The former theme identified that retention in trials does not seem to be given equal weight to recruitment, which is echoed by the dearth of methodology research on retention in favour of recruitment [4–6]. This imbalance appears to be reflected in the training offered to trial staff, with an emphasis on assessing medical eligibility and reaching suitable benchmarks for ethical consent, but perhaps at the expense of practical considerations that promote retention. In particular, staff that may be isolated from the day-to-day practices of follow-up due to trial design could underestimate the impact of their contributions to retention. Opportunities for recruiters to contribute positively to the probability someone is retained may be neglected in favour of the other aspects of the trial they have been trained to cover. More troubling are the behaviours detrimental to retention that are not addressed with alternative best practices. Such gaps in training may also explain why some of our participants demonstrated uncertainty, or outright pessimistic views, about their contributions to retention. Even for those staff tasked with follow-up, uncertainties on the effectiveness of their retention strategies demonstrates a potential lack of training and/or further reflects a lack of available evidence on effective retention strategies.

From interviews in this study, a sense that one’s behaviour is effective in contributing to retention is predicated on the weight attributed to factors outside the influence of their behaviour. Staff clearly demonstrated their motivations to work with their participants to keep them involved in the trial, but these efforts were moderated by the motivations and priorities of the participants themselves. Key elements to successful retention noted by our participants often described elements that could feasibly modulate the motivations and priorities of trial participants. The recurrent emphasis we witnessed on the quality and timing of retention-relevant communication, along with the quality of the relationships formed within trials, points to these interpersonal aspects of trial roles as potent potential levers for aligning discordant motivations and priorities throughout a trial. Gaining perspective on participant motivations and potential mechanisms for positively influencing these motivations has been identified by trial stakeholders (participants and staff) as important targets for further methodology research [3]. The perspectives of our interview participants address five of the top 10 priority questions set by this stakeholder group (Q1. What motivates a participant’s decision to complete a clinical trial; Q4; What are the best ways to encourage trial participants to complete the tasks (e.g., attend follow-up visits, complete questionnaires) required by the trial?; Q7. What are the most effective ways of collecting information from participants during a trial to improve retention?; Q8. How does a participant’s ongoing experience of the trial affect retention?; Q9. What information should trial teams communicate to potential trial participants to improve trial retention?) as seen in Table 5 [3].

**Table 5:** Mapping of findings to PRioRiTy 2 trial retention methodology targets [3]

Priority research question	This study’s related findings to research question (Belief statements from interviews)	How it may answer the question
1. What motivates a participant’s decision to complete a clinical trial?	When we maintain relationships with participants and emphasise the importance of their participation, it contributes to retention	Positive influence; perceived as necessary
	The relationships we establish and work to maintain with participants are crucial to retention	Positive influence; perceived as necessary
	I think there are changes to study documents that could better emphasise retention to both us as staff and participants	Neutral or relative influence; area for improvement
	I think participants’ competing priorities from ‘real life’ context can be a barrier to retention	Negative influence; perceived as detrimental
4. What are the best ways to encourage trial participants to complete the tasks (e.g., attend follow-up visits, complete questionnaires) required by the trial?	We should be able to establish and maintain effective relationships with participants over time	Positive influence; perceived as necessary
	When we set realistic expectations with participants about what trial participation involves, it leads to improved retention	Positive influence; perceived as necessary
	We try to accommodate our participants in order to improve retention	Positive influence; perceived as necessary
	I think flexible follow-up options help with retention	Positive influence; perceived as necessary
	I think questionnaires are confusing/cumbersome for participants	Negative influence; perceived as detrimental
	I think there are ways for us to deliver questionnaires to participants that would make it easier for them to complete and return them	Neutral or relative influence; area for improvement
7. What are the most effective ways of collecting information from participants during a trial to improve retention?	We try to accommodate our participants in order to improve retention	Positive influence; perceived as necessary
	I think flexible follow-up options help with retention	Positive influence; perceived as necessary
	I think questionnaires are confusing/cumbersome for participants	Negative influence; perceived as detrimental
	I think there are ways for us to deliver questionnaires to participants that would make it easier for them to complete and return them	Neutral or relative influence; area for improvement
8. How does a participant’s ongoing experience of the trial affect retention?	When we maintain relationships with participants and emphasise the importance of their participation, it contributes to retention	Positive influence; perceived as necessary
	You need to be able to set and manage expectations about follow-up with participants	Positive influence; perceived as necessary
	It feels like our participants’ motivation to be in our trial, not necessarily how motivated we are, predicts whether we can retain	Neutral or relative influence; area for improvement
	I am concerned about participants feeling coerced into a trial and I work to remain impartial in my conversations	Neutral or relative influence; area for improvement
9. What information should trial teams communicate to potential trial participants to improve trial retention?	We should be able to communicate what participation involves early and explicitly	Positive influence; perceived as necessary
	When we set realistic expectations with participants about what trial participation involves, it leads to improved retention	Positive influence; perceived as necessary
	It is necessary that I discuss follow-up	Positive influence; perceived as necessary
	I think consent is only valid if participants have received an adequate amount of information about follow-up	Positive influence; perceived as necessary
	My role as a recruiter is to be transparent with participants about what study participation actually involves	Positive influence; perceived as necessary
	You need to be able to set and manage expectations about follow-up with participants	Positive influence; perceived as necessary

For many trials, there is complex interplay between groups of individuals involved in delivery. Recruiters, regardless of their later roles, are a participant’s introduction to the social networks involved in running trials. They need to set realistic expectations about risks, potentially limited benefits, and commitments of time and effort that are required by the trial in order to be a participant. Recruiters have a restricted window to set these expectations but need to do so impactfully, particularly if they do not have ongoing contact with participants. It is here that recruiters should seek to highlight follow-up in a way that it reframes the sometimes-abstract trial outcomes to the perspective of the participant. Staff involved in follow-up could employ a complementary suite of behaviours supporting retention, providing ongoing management of a participant’s retention behaviours. Examples of such behaviours could include debriefing participants after appointments, discussing particulars of the next visit, or reminding them of the accommodations and support that the trial team can offer. Oftentimes, it was mentioned that this support required is based on relational aspects. The relationships between staff and participants rely on the social and communicative behaviours exercised by staff. Several staff mentioned that the relationships they form with their participants serves to reinforce their own retention behaviours. These results parallel findings on what relational aspects of trials are important to participants [32]. As our interview participants seemed to place a premium on the ability to establish and maintain effective relationships (echoed by other research with staff [4]), a perceived strain or breakdown in these relationships could have implications on staffs’ confidence in their roles. The knock-on effects from this disturbed confidence have the potential to exert detrimental effects at several points along the trial retention pathway. Taken together, our results demonstrate a need to further explore the impact of communication practices and relational factors within the context of trial staffs’ behaviour. Importantly, the interaction between this behaviour and their participants’ retention behaviour is a pivotal area to consider for possible intervention design. Pursuing such avenues towards effective interventions that address issues within retention will serve to fill gaps seen within the current evidence base. That gap is highlighted in the recent Cochrane review of retention interventions which identified very few existing interventions with even moderate-certainty evidence (4/70 interventions) and primarily low effect size (1–7% improvement in retention outcomes) [refworks crashed]. Overwhelmingly, those interventions (68/70) sought to intervene on participants which further leaves open the possibility that interventions aimed at staff may prove to be a severely undeveloped resource of moderate to high rates of improvement in retention outcomes.

### Strengths and limitations

This study shares many of the same strengths and limitations of previous interview studies using the TDF. The structured approach to topic guide design and coding of data is useful in its systematic exploration of behavioural domains known to be relevant in understanding behaviours [30]. However, there are criticisms that this approach may restrict topics of conversation important to interview participants and the predominantly deductive nature of analysis prohibits including results that do not “fit” within these domains [33]. Efforts have been made in this study to utilise the topic guide flexibly to facilitate a more natural flow of conversation. Open-ended questions outside the TDF domains were also included to prompt interview participants to discuss topics not already addressed by the topic guide. Similarly, the analysis was carried out in both deductive and inductive phases to capture as much pertinent data as possible. Our studies made use of behavioural specification guidelines when identifying the target behaviours for each interview set to facilitate efforts in interpretation and replication by others. However, interview participants did not always cooperate with our intended defined boundaries of behaviour when discussing their experiences. As such, an inclusive approach to the target behaviour of “retention” was adopted to make use of such data. Data was also generated within our specific target behaviour of “retention communication at consent” to allow analysis within this more narrowly defined context. We believe this two-pronged approach to behavioural specification in analysis has allowed for a more comprehensive approach. This is particularly important in the context of these studies as they aimed to explore a relatively unknown area of methodology research.

A potential limitation of the data set could be that the main author (TC) was not involved in the planning or conduct of the first set of interviews. However, as other members of the research team were directly involved with the implementation of this prior study, there is a marked consistency in the conduct and quality of both studies, complemented by integrated, independent analysis of one set of data. The interviewer in that (RN) study was consulted prior to the second interview set was conducted and was available throughout for questions regarding the context of the first interview set [22, 27]. Topic guides and coding guides for the first interview study served as references for the development of the analogous documents in the second interview study, which further aligned their conduct.

Our study sample, while notably diverse, is still subject to the limitations of self-selection bias and a relatively small sample size. Trials involved in this study, and the staff recruited, may be comprised of individuals who are particularly motivated in their roles and so may not be representative of the larger trials community. In addition, as our host trials were solely pragmatic effectiveness trials, we cannot speak to the possible reasons behind non-retention in other types of trials, such as early phase trials where the influences on staff involved in recruitment discussing retention will likely differ. Future work on the feasibility and acceptability of any interventions generated from these results will look to counteract this by soliciting the opinions of those outside our sample. However, a strength of our study sample is the breadth of experience cited by our participants and in the diversity of the trials that they have gained that experience from. Ideally, this diversity will confer a generalisability of our results and a wider applicability of the interventions produced.

## Conclusions

The themes generated in our interview study present the barriers and facilitators to retention from a breadth of roles and levels of experience in trials. As the consequences of poor retention present a threat to the validity of any trial, our research aimed to elucidate the complex mechanisms underlying its success or failure. Key findings add detail on the behavioural impact of preoccupation towards recruitment, elaboration on the roles staff believe they play towards retention, and, most importantly, perspective on how staff look to excel in those roles through successful communication and rapport development. Future efforts should focus on intervention development based on these findings to improve how trial staff involved in recruitment enable discussions about trial retention during informed consent.

## Supplementary Information

**Additional file 1.** Topic guides for interviews. Final versions of TDF topic guides used in interviews is provided. Two topic guides are available in the file. The first is the topic guide for the retention staff interviews, the second is for the recruitment staff interviews.

**Additional file2.** COREQchecklist. Completed COREQ checklist for the manuscript.

**Additional file 3.** Additional detailson host trials. Table giving additional details on the five host trials sampledfrom for the recruitment staff interviews. Data is populated to provide contexton the trial outcomes and delegation of staff responsibilities.

## Data Availability

The datasets used and/or analysed during the current study are available from the corresponding author on reasonable request.

## Abbreviations

AACTT: Action, Actor, Context, Target, Time
A&E: Accident and emergency
APEASE: Acceptability, Practicability, Effectiveness, Affordability, Side-effects, and Equity
CTIMP: Clinical Trial of Investigational Medicinal Products
ECG: Electrocardiogram
GCP: Good clinical practice
GP: General practitioner
PIL: Participant (patient) information leaflet
PROM: Patient-reported outcome measure
NHS: National Health Service
NIHR: National Institute for Health Research
SPIRIT: Standard Protocol Items: Recommendations for Interventional Trials
SWAT: Study within a trial
TDF: Theoretical Domains Framework
