# Supplementary material for: What influences communication about retention in randomised trials: a multi-trial, theory-based analysis exploring trial staff perspectives
Source: BMC Med Res Methodol. 2022 Aug 25;22:231. doi: 10.1186/s12874-022-01708-4 (PMC9404662; doi:10.1186/s12874-022-01708-4)
Supplement: Supplementary file 1 — Additional file 1. Topic guides for interviews. Final versions of TDF topic guides used in interviews is provided. Two topic guides are available in the file. The first is the topic guide for the retention staff interviews, the second is for the recruitment staff interviews. [file 12874_2022_1708_MOESM1_ESM.docx]

Retention staff topic guide

**STEER: Systematic Techniques to Enhance REtention in RCTs**

Firstly, thank you for agreeing to take part in this interview study and agreeing to speak with me today.

The overall aim of our study is to inform how to improve retention in trials.

- I am Rumana Newlands, a researcher at the UoA. As part of my work, I am conducting interviews with trial staff to explore their experiences of retaining participants in trials. I’d like to know the strategies that you may have used to deal with retention challenges. By retention challenges, I mean, issues with loss to follow-up due to no response to questionnaires (other methods of remote data collection) or patients not attending clinic for follow-up data collection as part of the trial.

-I want to emphasize that there are no right or wrong answers and we’re not interested in making any judgements, rather, we’re keen to hear about your views and experiences with retaining patients and any challenges you have faced so that we can best help future trials to retain patients.

-What we discuss today will remain confidential and we will use anonymised transcripts for the research purpose and its dissemination. Your name will not be mentioned anywhere nor will your line manager or the trial team ever know what you specifically say.

- What you say will not be used to make any judgement about you or what happened previously. Most of the chats that I’ve had with other people have taken around 20-40 minutes; is that ok?

- I would like to audio record this interview so that we have an accurate record of it and I don’t have to rely on my memory. Please confirm that you are happy for the interview to be conducted and recorded.

-SWITCH ON RECORDER AND

-READ OUT PARTICIPANT NUMBER

***For the purpose of recording could you please confirm again that you are happy to be interviewed today and happy to record our conversations?***

Thank you. Any questions before we start?

**Topic guide- STAFF** [**behaviour of interest** = Any actions and non-actions of the trial staff that may have influenced non-retention of trial participants

**TACT-A specified behaviour** [Target= trial participants; Actor= Trial staff, Action= actions/non-actions that influenced non retention Context= various e.g. trial office (on the phone, by email, web-based applications, etc), clinic (i.e. face-to-face); Time= dependent on trial follow-up time points]

*To start with can I ask you a few questions about your back ground?*

| **Domains** | **Core questions** | **Possible prompts** |
| --- | --- | --- |
| **Opening questions** ( personal and trial background) | | |
| **Background about trial/staff** | 1. How do you identify your gender? 2. What is your job title? 3. How long have you been a (job title)? |  |
| **Opening questions** | 1. Can you tell me what your general views are on retention of participants in trials? 2. What are the factors that lead to retention in RCTs? 3. What are the factors that lead to loss of follow-up (across trials)?   *(Why do you think it can be difficult to keep participants in trials?)*   1. Can you give me an idea of some situations where it becomes difficult to retain participants in trials? | |
| 1. **Knowledge**   [an awareness of the existence of something]  (knowledge about the behaviours involved in being a trial staff)  TRIAL SPECIFIC | 1. What’s the retention rate (so far) of the … trial? 2. How do you follow-up participants for … trial? 3. How many follow-up points? 4. What type of queries do you normally get once the trial is ongoing? | -If using Questionnaires ask- what modes do you use i.e. paper in the post/email/online/text based/ apps etc   - Any queries related to trial activities- Q/clinic appointments? |
| 1. **Goals**   [Mental representations of outcomes or end states that an individual wants to achieve]  GENERAL / TRIAL SPECIFIC | 1. How important you feel it is to retain participants in trials (e.g. returning Qs or attending clinics)? 2. Did/do you have any particular goal/target for  - Qs return rate - clinic attendance rate?  1. Where does retaining trial participants fit in your priorities?   *(By fitting in your priorities, I mean, how important is this (trying to retain participants) compared to other things you do at work?)*   1. How important is this compared to other things you are doing? 2. What are your other priorities? | -How many is it?  -Who sets it? |
| 1. **Intentions**   [A conscious decision to perform a behaviour or a resolve to act in a certain way] | 1. How motivated are you to retain participants in trials? | - What **reduced** your motivation?   (e.g. any negative experiences?)   - What **increased** your motivation?   -Why do you think that? |
| 1. **Skills**   [an ability or proficiency] | 1. What do you think, trial staff, need to be able to do    to improve questionnaire responses   to improve clinic attendance   Other activities (check PIL)?   To keep participants in the trial until the end   1. Have you had any training or workshops related to retaining participants in the trial? 2. How do you feel contacting participants if they missed a follow-up? | - What skills are required to retain participants in the trial?  - What are they?  - What else?  -What do you mean by that?   - *ask if did not mention previously*- how do they contact participants (by email/phone/text?) |
| 1. **Beliefs about capabilities**   [acceptance of the truth, reality or validity about an ability, talent or facility that a person can put to constructive use] | 1. How confident are you that you could retain participants in trials?   - ***If low:*** what made you less confident? Is there anything that would increase your confidence?  -***If high-*** What made you feel more confident?   1. What (other) **problem/difficulties** do you encounter for retaining participants in the trials? *(might have answered)* 2. How much do that influence whether you try to retain participants in the trial? 3. What would have made it **easier** to retain participants? 4. How much would that influence whether you try to retain participants in the trial? | - Can you tell me a bit more about it please?  -Anything else? |
| 1. **Memory, attention & decision processes**   (The ability to retain information, focus selectively on aspects of the environment and choose between two or more alternatives)  *(relate to their non-retention behaviour)* | 1. Is it possible to forget to follow/contact participants (sometimes) if they missed a clinic appointment or did not return a questionnaire?   - How can this be avoided? (code under B. regulation) | -**What** happened?  -What else? |
| 1. **Environmental context and resources**   [any circumstance of a person’s situation or environment that encourages/discourages] | 1. Are there any aspects of the work environment that make it ***easier*** for you to retain participants in the trial/prevent loss to follow-up? 2. Are there any aspects of the work environment that make it ***difficult*** for you to retain participants in the trial/prevent loss to follow-up? 3. Do you believe there are any barriers to patients’ participation in the trial? 4. Among all the issues you have mentioned, which of these had the **biggest impact** on … trial (in relation to retention)? 5. What do you think could help overcome these barriers? *(behavioural regulation but keep here now)* 6. What resources are needed for you to be able to retain participants in trials? 7. To what extent are these resources available in your unit/office? 8. How will it impact your ability to retain trial participants? | -What else?  -Are there any competing tasks or time constraints that influence whether you attempt to retain a participant in the trial or not? *(only if they struggle)*  -Any patient/colleague factors?  -Any other resource issues?  - Any issues raised by participants related to completing a questionnaire or attending **a** clinic appointment?  e.g. lay out-difficult to follow  length/time to complete/issues with travelling-venue   - Anything else?   -  -Can you suggest any potential solutions? |
| 1. **Emotion**   [a complex reaction pattern, involving experimental, behavioural and psychological elements by which the individual attempts to deal with a personally significant matter or event] | This may be bit of an odd question, but bear with me.   1. What sort of feelings come to mind when you think of retaining participants in the trial? 2. How do you think emotions, or tense situations in the unit/office affect your work related to retaining participants? 3. Can you describe me the sorts of emotions/feelings that you might have experienced while contacting non-responders   (to complete a questionnaire/rearrange clinic appointment)? | -contacting non-responders  -chasing after  *-*what upset you?  -why you were worried?  -Does patients’ emotions affect your approach in completing a questionnaire/re-arranging an appointment?  What make you feel uncomfortable?  -What else?  -Can you tell me more, if that’s okay with you? |
| 1. **Social influences**   [Those interpersonal processes that can cause individuals to change their thoughts, feelings or behaviours] | 1. Whose opinion (views) is important to you when deciding to try and retain participants? 2. How does views of other colleagues affect your approach in retaining participants? 3. Do you discuss participant retention issues with anyone within your trial team? | - Whose views impact most on whether you approach participants for retention? Why?  - Do other colleagues support you – to retain participants?  - How does that impact on whether you try and retain?  -How did the discussion go in the past?  -Do they support you? In what way?  -What do you do then? |
| 1. **Beliefs about consequences**   [acceptance of the truth, reality or validity about outcomes of a behaviour in a given situation] | *The next few Qs I am going to ask is about consequences and understanding your views about these situations. Please do not feel that you are being judged for any situations as such*   1. What are the **upsides/downsides** of *(follow Qs from next domain)*  - Retaining participants in a trial until the end? - When participants don’t return questionnaires? - When participants don’t attend clinic appointments?  1. Do you feel that you have already made a difference to the … trial (by retaining participants)?   *(follow Qs from next domain)* | -What would happen if you were unable to successfully retain participants to … trial?  - Are there any benefits and downsides   - to the trial/to NHS/future patients? - what else?   - In what way? |
| 1. **Optimism**   [The confidence that things will happen for the best or that desired goals will be attained] | 1. Can you tell me more about … (*If it feels appropriate based on responses under above domain)?* 2. Overall, did you expect participants to retain in … trial?   -Why were you ***so*** optimistic?  -Why were you ***not so*** optimistic? | **How** this would benefit/disadvantage …?   - You/the trial/future patients |
| 1. **Social professional role & identity**   (for patients it’s all about personal identity) | 1. To what extent do you see retaining participants in trials as part of your current professional role?      1. If No, is it anyone else’s job? Whose should it be? 2. Why? How should they be involved? 3. Who deals with monitoring retention/non-retention of trial participants? | - Can you please tell me a bit about your role in relation to the … trial?  - What are these roles?  -Should anyone else be involved? Who? |
| 1. **Behavioural regulation**   [Anything aimed at managing or changing objectively observed or measured actions]  **All non-retainers** | 1. When do you perceive loss to follow-up/non-retention to be a problem? 2. How do you deal with the situation then?   *(do you send reminders/how many/how/how often?)*   1. How do you decide which strategies to improve follow-up to implement? 2. What strategies did you implement and how to retain participants in … trial? 3. What strategies to increase **a)** questionnaire response or **b)** clinic attendance have been successful for you in trials that you have worked on? 4. What strategies have been unsuccessful? 5. Is there anything that you would alter or do differently concerning how you act on retaining participants in the trial? 6. What do you think is needed to ensure that appropriate strategies (approaches) are used to retain participants in the trial? 7. Based on your experience, do you have any suggestions on how trialists could retain participants in trials? 8. What do you think about using various (*if not mentioned earlier in the interview*) 9. Communication strategies:  - email/telephone/text messages for staying in touch - letters signed by different study personnel - type of delivery (1^st^ or 2^nd^ class)/recorded delivery/ - type of envelope used for response?  1. Methodological strategies: blind versus un blind trials 2. Different length of questionnaire: short versus long | - How is loss to follow-up **monitored?** - When should it be done/tackled? - By whom?   - What are the reasons given, if any, regarding not returning a questionnaire or not attending a clinic?  - **Why** do you think these have worked?  -What other evidence are you aware of, or do you use?  - What else?  -**Why** have these not worked?    - What would you do differently?    -At the beginning or when the trial is ongoing?  -What could be the advantage and disadvantage of using this strategy? |
| 1. **Reinforcement**   [Increasing the probability of a response by arranging a dependent relationship or contingency, between the response and a given stimulus]  (TISU) | 1. What does encourage/discourage you to retain trial participants? 2. Has anything in the past encouraged/discouraged you to retain participants in the trial? 3. What previous experiences were **rewarding** to you to retain…? 4. Are there any **negative experiences** that may discourage you to try and retain participants? 5. What sort of rewards would be acceptable to you? 6. How about incentives to either participants or trialists e.g. gifts pen, coat pins, monetary incentives, offers of incentives, vouchers | (e.g. like recognition from peers/-From whom?)  -Anything else?  -What would be the advantages and disadvantages of using this strategy? |

**Interview close out questions**

- Is there anything that you would like to add about your views and experience of retaining participants in …trial?

- Any other factors that you think might be important that we haven’t covered?

-Is it ok if I contact you if there are any points of clarification?

-Although there is no obligation, would you like to be contacted for any future linked research (e.g. focus group meetings)?

Thanks again for the chat. What you have said is confidential and no judgement will be made but your discussion will definitely help us for developing strategies towards improving retention of trial participants.

**Thank you**

Recruitment staff topic guide

Inform participants that:

Thank you for agreeing to participate in this interview. The general aim of the study is to help us understand more about the experience of recruiting participants into trials. Specifically, I’d like to ask you about some of the information that you may communicate to participants that may be useful in keeping them on study until the end. I have a number of questions I’m going to ask you - and I’d like you to give them some thought and answer frankly. We are interested in your experiences and views so there are no right or wrong answers.

Do you have any questions for me before we start?

First, I wanted to agree on the definition of retention that we are going to be using today. So firstly, could you tell me what you think of as being retention and the sorts of things associated with it?

Retention is broadly going to be two things: 1. Participants completing study follow-up throughout the study and 2. Completing the primary end point (s) of the study and ending their participation by doing so. Now, some examples of how retention might come up when you are speaking with a potential participants are going to include: them understanding the study schedule and their time commitments throughout the study until its completion, completing study procedures that are key to collecting outcome data (e.g. attending clinic/phone visits, completing/returning questionnaires), their right to withdraw and how to go about doing so, whether they can do things like stop assigned treatment but still participate in follow up for data collection, and generally why their commitment is important (e.g. the value of complete data, the importance of their contributions, issues that arise for trials if they are not retained). As for when these conversations occur, we are primarily focused on the conversations that are happening immediately before participants sign consent. However, we realise that there might be multiple points that you or a potential participant reach out and have conversations about the study. So, we do want to hear about those but we are not so much interested in the documents or emails that you might send, just the verbal communication.

Right, so if you can try to keep those criteria in mind but if anything needs clarification now or throughout, please do ask.

------------------------------------------------------------------------------------------------------------------------------

1. What is your job title?

2. How long have you been a (job title)?

3. How many trials have you been/are you currently involved in as a recruiter?

4. Can you tell me about your role in recruitment of participants -

a. pertaining to [insert trial]?

b. regarding your current trial (s)?

c. If not currently involved as a recruiter: When was the last time you were involved in recruitment and what was your role?

d. If also involved in follow-up, repeat prompts above as relevant.

| Domain | Question |  |
| --- | --- | --- |
| Social/professional role and identity  (A coherent set of behaviours and displayed personal qualities of an individual in a social or work setting) | How does talking to potential participants about follow-up and what completing the study entails fit within your role? | Asked  Answered  Answered (response to other Qs)  Return |
| Knowledge  (An awareness of the existence of something) | Okay, so now I’d like you to imagine that I’m a potential participant for [insert trial]. Could you go through key points that you’d normally present during the consent process when discussing your trial?  Prompt: Now thinking back to our definition of retention, is there anything else you normally present during these discussions that you feel falls under that definition? | Asked  Answered  Answered (response to other Qs)  Return |
| Intentions  (A conscious decision to perform a behaviour or a resolve to act in a certain way) | Do you always intend to discuss retention when you talk to participants?  Prompt: Why/why not?  Prompt: Does this vary or has this changed over time? | Asked  Answered  Answered (response to other Qs)  Return |
| Goals  (Mental representations of outcomes or end states that an individual wants to achieve) | Is discussing follow-up and what completing the study entails with participants a priority for you?  Prompt: what are other priorities during the recruitment discussion? | Asked  Answered  Answered (response to other Qs)  Return |
| Skills  (An ability or proficiency acquired through practice) | Have you ever received training on how to discuss follow-up and what completing the study entails to potential participants?  Prompt re: GCP: So, within the GCP training, did it cover topics related to retention? | Asked  Answered  Answered (response to other Qs)  Return |
| Beliefs about capabilities  (Acceptance of the truth, reality or validity about an ability, talent or facility that a person can put to constructive use) | How confident are you in your ability to discuss with potential participants what is expected of them in your trial and the importance of completing follow-up? | Asked  Answered  Answered (response to other Qs)  Return |
|  | Do you feel you have any influence on whether someone remains on your trial? | Asked  Answered  Answered (response to other Qs)  Return |
| Optimism  (The confidence that things will happen for the best or that desired goals will be attained) | Do you feel that discussing completing study follow-up with potential participants at the point of recruitment makes a difference to retention overall?  Prompt: Optimistic/Pessimistic; Why/Why not? | Asked  Answered  Answered (response to other Qs)  Return |
| Beliefs about Consequences  (Acceptance of the truth, reality, or validity about outcomes of a behaviour in a given situation) | What are the drawbacks of discussing follow-up and what completing the study entails with potential participants?  What are the benefits? | Asked  Answered  Answered (response to other Qs)  Return |
| Reinforcement  (Increasing the probability of a response by arranging a dependent relationship, or contingency, between the response and a given stimulus) | Are there any incentives for you to talk about follow-up and what completing the study entails?  Prompt: Personal, team, rewards  Do you receive any feedback about your recruitment discussions? | Asked  Answered  Answered (response to other Qs)  Return |
| Social influences  (Those interpersonal processes that can cause individuals to change their thoughts, feelings, or behaviours) | Do other recruiters/members of trial staff bring up follow-up and what completing the study entails during their recruitment discussions or in meetings? | Asked  Answered  Answered (response to other Qs)  Return |
|  | Would any other team members influence whether or not you discuss follow-up and what completing the study entails with potential participants?  Prompt: who else; other clinicians; medical staff/nurses and doctors | Asked  Answered  Answered (response to other Qs)  Return |
| Environmental context and resources  (Any circumstance of a person’s situation or environment that discourages or encourages the development of skills and abilities, independence, social competence and adaptive behaviour) | Are there any factors in your work environment that affect whether you discuss follow-up and what completing the study entails? | Asked  Answered  Answered (response to other Qs)  Return |
|  | Do you use any protocols, policies, or guidelines to guide your discussions for trial recruitment?  Prompt: And specifically, to focus on retention? | Asked  Answered  Answered (response to other Qs)  Return |
| Behavioural regulation  (Anything aimed at managing or changing objectively observed or measured actions) | Are there ways of working that help you when having recruitment discussions?  Prompt: Do you have a checklist or something similar that you’ve made of what information you provide during recruitment discussions? | Asked  Answered  Answered (response to other Qs)  Return |
| Memory, attention, and decision processes  (The ability to retain information, focus selectively on aspects of the environment and choose between two or more alternatives) | Is remembering to talk about follow-up and what completing the study entails difficult or easy to do? | Asked  Answered  Answered (response to other Qs)  Return |
| Emotion  (A complex reaction pattern, involving experiential, behavioural, and physiological elements, by which the individual attempts to deal with a personally significant matter or event) | How do you feel when discussing follow-up and what completing the study entails with potential participants?  Prompt: frustrating/satisfying, stressful/not | Asked  Answered  Answered (response to other Qs)  Return |

(If time allows) Finally, just some background information.

1. What best describes your gender identity?
   1. Prompt: female, male, prefer to self-describe, or prefer not to say?
2. What ethnicity do you identify as?

This project is looking to use the results from these interviews to design strategies to address some of the issues to retention. We’re going to be doing that through something called a co-design exercise, which is like a focus group, where we meet to discuss some of the practical ways that we can implement the lessons learned here. We’d really value your input for that if you’d be willing to let us reach back out to you once we schedule those groups. It would probably be next spring and would be a one-off session over a couple hours, but we’d have more details closer to the event. Are you willing to be contacted about participating in that?

That’s all the questions I have for you, is there something we’ve not covered that is important you’d like to say or expand on?

Thank you very much for your time.
