# Supplementary material for: What influences communication about retention in randomised trials: a multi-trial, theory-based analysis exploring trial staff perspectives
Source: BMC Med Res Methodol. 2022 Aug 25;22:231. doi: 10.1186/s12874-022-01708-4 (PMC9404662; doi:10.1186/s12874-022-01708-4)
Supplement: Supplementary file 2 — Additional file2. COREQchecklist. Completed COREQ checklist for the manuscript. [file 12874_2022_1708_MOESM2_ESM.docx]

**Domain 1: Research team and reflexivity**

| Personal Characteristics |  |  |  |
| --- | --- | --- | --- |
| 1.  Interviewer/facilitator | | Which author/s conducted the interview or focus group? | TC |
| 2.  Credentials | | What were the researcher's credentials? *E.g. PhD, MD* | TC - M.Sc.; KG, ED, HM – PhD |
| 3.  Occupation | | What was their occupation at the time of the study? | TC – PhD student; KG - Director of the Health Care Assessment Programme and Reader;  ED - Research Fellow and Health Psychologist; HM – Lecturer (Scholarship) |
| 4.  Gender | | Was the researcher male or female? | TC – Male; KG, ED, HM – Female |
| 5.  Experience and training | | What experience or training did the researcher have? | TC – workshops/training events in qualitative research and interviewing, training school in behavioural methodology through UCL; KG, ED, HM – lines 182-183 |
| Relationship with participants | |  |  |
| 6.  Relationship established | | Was a relationship established prior to study commencement? | No relationships were established with interview participants prior to study commencement. |
| 7.  Participant knowledge of the interviewer | | What did the participants know about the researcher? e*.g. personal goals, reasons for doing the research* | All participants had contact with TC while being recruited to the interviews and were introduced to the objectives of the overall project and the purpose of the interviews. |
| 8.  Interviewer characteristics | | What characteristics were reported about the interviewer/facilitator? e.g. *Bias, assumptions, reasons and interests in the research topic* | TC introduced all interview participants to his personal research interests and reasons for studying the topic, along with his personal history of working with clinical trials. It was emphasised that these experiences were within the US vs. the UK and so all assumptions held were subject to the need for further explanation by the participants. |
| **Domain 2: study design** | | |  |
| Theoretical framework | | |  |
| 9.  Methodological orientation and Theory | | What methodological orientation was stated to underpin the study? *e.g. grounded theory, discourse analysis, ethnography, phenomenology, content analysis* | The study was explained to be underpinned by the Theoretical Domains Framework. Participants were briefed on the semi-structured nature of the interviews and the use of a topic guide to conduct interviews. It was explained that the using the Framework to understand various aspects of behaviour would sometimes cause questions to appear strange or repetitive. Participants were encouraged to ask for explanations of each question as well as feeling free to answer questions however they deemed appropriate.  Pgs. 9-11 |
| Participant selection | | |  |
| 10.  Sampling | | How were participants selected? *e.g. purposive, convenience, consecutive, snowball* | Pgs. 7-9 |
| 11.  Method of approach | | How were participants approached? e*.g. face-to-face, telephone, mail, email* | Pg. 9 |
| 12.  Sample size | | How many participants were in the study? | Pgs. 11-13 |
| 13.  Non-participation | | How many people refused to participate or dropped out? Reasons? | All participants that volunteered to participate completed the interview. |
| Setting | | |  |
| 14.  Setting of data collection | | Where was the data collected? e*.g. home, clinic, workplace* | Pgs. 8-9 |
| 15.  Presence of non-participants | | Was anyone else present besides the participants and researchers? | No. |
| 16.  Description of sample | | What are the important characteristics of the sample? *e.g. demographic data, date* | Pgs. 7-8, 11-13 |
| Data collection | |  |  |
| 17.  Interview guide | | Were questions, prompts, guides provided by the authors? Was it pilot tested? | Pgs. 9-10, Additional File 1 |
| 18.  Repeat interviews | | Were repeat interviews carried out? If yes, how many? | No. |
| 19.  Audio/visual recording | | Did the research use audio or visual recording to collect the data? | Pgs. 8-9 |
| 20.  Field notes | | Were field notes made during and/or after the interview or focus group? | TC made notes following each interview. |
| 21.  Duration | | What was the duration of the interviews or focus group? | 30 minutes – 1 hour |
| 22.  Data saturation | | Was data saturation discussed? | No, data collection occurred during a pre-specified timeframe, with interviews concluding once that time had elapsed. |
| 23.  Transcripts returned | | Were transcripts returned to participants for comment and/or correction? | No. |
| **Domain 3: analysis and findings** | |  |  |
| Data analysis | |  |  |
| 24.  Number of data coders | | How many data coders coded the data? | Pg. 10 |
| 25.  Description of the coding tree | | Did authors provide a description of the coding tree? | Pgs. 10-11 |
| 26.  Derivation of themes | | Were themes identified in advance or derived from the data? | Pgs. 10-11 |
| 27.  Software | | What software, if applicable, was used to manage the data? | Pgs. 10 |
| 28.  Participant checking | | Did participants provide feedback on the findings? | No. |
| Reporting | |  |  |
| 29.  Quotations presented | | Were participant quotations presented to illustrate the themes / findings? Was each quotation identified? e*.g. participant number* | Tables 3 and 4 |
| 30.  Data and findings consistent | | Was there consistency between the data presented and the findings? | Yes. |
| 31.  Clarity of major themes | | Were major themes clearly presented in the findings? | Pgs. 13-22 |
| 32.  Clarity of minor themes | | Is there a description of diverse cases or discussion of minor themes? | Pgs. 13-22 |
