# Supplementary material for: What influences communication about retention in randomised trials: a multi-trial, theory-based analysis exploring trial staff perspectives
Source: BMC Med Res Methodol. 2022 Aug 25;22:231. doi: 10.1186/s12874-022-01708-4 (PMC9404662; doi:10.1186/s12874-022-01708-4)
Supplement: Supplementary file 3 — Additional file 3. Additional detailson host trials. Table giving additional details on the five host trials sampledfrom for the recruitment staff interviews. Data is populated to provide contexton the trial outcomes and delegation of staff responsibilities. [file 12874_2022_1708_MOESM3_ESM.docx]

Additional File 3: Trial outcomes and staff responsible for collecting data leading to said outcomes in recruitment staff interviews; aTrial is a pilot and did not differentiate between primary/secondary outcomes in the way that full-scale trials did; b Measures on

| **Trial outcome(s)** | | **Timepoints** | **Staff responsible** | **Participant involvement** |
| --- | --- | --- | --- | --- |
| Trial 1 | | | | |
| Primary | Participant reported functional score on standardised measure | 4 months post treatment | Central trial staff | Remote follow-up via email and/or SMS |
| Secondary | Differences between intervention groups in function, complication risk, and health-related quality of life | 8 weeks, 4 months, 12 months, annually until 5 years post-treatment | Central and local trial staff | Remote follow-up via email and/or SMS |
|  | Economic evaluation: medical record and resource use questionnaire | 6 weeks and 12 months | Central trial staff | 6 week: Attendance at clinic (standard of care visit)  12 month: Remote |
| Trial 2 | | | | |
| Primary | Comparison of patient reported success rates following treatment | 15 months post randomisation | Central trial staff | post, e-mail, web based and SMS |
|  | Cost effectiveness of intervention vs standard care | 6 and 15 months post randomisation | Central and local trial staff | post, e-mail, web based and SMS |
| Secondary | Assess primary outcomes in specific population subgroups | 3, 6, and 15 months post randomisation | Central trial staff | post, e-mail, web based and SMS |
|  | Explore participant attitudes/experiences in both treatments | 3-6 months post treatment | Central trial staff | Face to face participant interviews |
| Trial 3^a^ | | | | |
| Primary | Patient reported outcomes of function, health, and well-being; | 4 weeks post-intervention, 1 week prior to surgery,  3 months post-surgery | Central and local trial staff | 4 weeks: Phone visit;  Others: returned via post or completed over phone |
|  | Feasibility/  acceptability of trial design/intervention | Not reported | Central trial staff | Participant interviews |
| Trial 4 | | | | |
| Primary | Clinical effectiveness of pharmaceutical intervention vs placebo, measured by standardised symptom scale | 12 weeks | Local trial staff | Clinic or remote visit |
| Secondary | Comparison of pharmaceutical intervention to other active treatment | 6, 12, 24, and 52 weeks | Local trial staff | Clinic or remote visit (Weeks 24 and 52, postal questionnaire) |
|  | Cost-effectiveness/safety of pharmaceutical intervention when added to standard care | 6, 12, 24, and 52 weeks | Local trial staff | Clinic or remote visit (Weeks 24 and 52, postal questionnaire) |
| Trial 5 | | | | |
| Primary | Hospital admission for treatment | Within 18 months of randomisation | Local trial staff | N/A |
|  | Complications associated with treatment | During and immediately following treatment | Local trial staff | N/A |
|  | Complications associated with intervention procedure | During and immediately following intervention | Local trial staff | N/A |
| Secondary | Health-related quality of life^b^ | 3, 6, 12, and 18 months post randomisation | Central trial staff | Post or online questionnaire |
|  | Core outcome set related to treatment | 90 days post treatment | Central and local trial staff | N/A |
|  | NHS resource utilisation | 18 months of randomisation | Central and local trial staff | N/A |
